# Viral RNA Load in Mildly Symptomatic and Asymptomatic Children with COVID-19, Seoul, South Korea

**DOI:** 10.3201/eid2610.202449

**Published:** 2020-10

**Authors:** Mi Seon Han, Moon-Woo Seong, Namhee Kim, Sue Shin, Sung Im Cho, Hyunwoong Park, Taek Soo Kim, Sung Sup Park, Eun Hwa Choi

**Affiliations:** Seoul Metropolitan Government–Seoul National University Boramae Medical Center, Seoul, South Korea (M.S. Han, N. Kim, S. Shin, H. Park);; Seoul National University College of Medicine, Seoul (M.-W. Seong, S. Shin, H. Park, T.S. Kim, S.S. Park, E.H. Choi);; Seoul National University Hospital, Seoul (M.-W. Seong, S.I. Cho, T.S. Kim, S.S. Park);; Seoul National University Children’s Hospital, Seoul (E.H. Choi)

**Keywords:** SARS-CoV-2, viral load, children, COVID-19, feces, saliva, Seoul, South Korea, viruses, respiratory infections, coronavirus, coronavirus disease, COVID-19, severe acute respiratory syndrome coronavirus 2, zoonoses

## Abstract

Along with positive SARS-CoV-2 RNA in nasopharyngeal swabs, viral RNA was detectable at high concentration for >3 weeks in fecal samples from 12 mildly symptomatic and asymptomatic children with COVID-19 in Seoul, South Korea. Saliva also tested positive during the early phase of infection. If proven infectious, feces and saliva could serve as transmission sources.

In the current pandemic of coronavirus disease (COVID-19), detecting severe acute respiratory syndrome coronavirus 2 (SARS-CoV-2) in children suspected of having the disease is essential for both infection control and establishing a definite causal relationship in unprecedented cases (*1*,*2*). However, efforts are hindered by negative SARS-CoV-2 test results for respiratory specimens and possible cross-reactivity with other coronaviruses among seropositive cases (*2*,*3*). Little is known about the value of various samples other than nasopharyngeal or oropharyngeal swab specimens in diagnosing COVID-19 and understanding the viral dynamics of SARS-CoV-2 in children. Virus RNA was persistently detected in rectal swab specimens in a previous study, although the infectiousness of the virus is unknown (*4*). We analyzed the viral RNA load kinetics of SARS-CoV-2 in various clinical specimens in children with COVID-19.

In South Korea, all confirmed case-patients, regardless of disease severity, must be isolated in hospitals or isolation facilities. For this study, we included all children <18 years of age who were confirmed to have COVID-19 by positive results for SARS-CoV-2 in combined nasopharyngeal and oropharyngeal swab specimens and who were hospitalized in Seoul Metropolitan Government–Seoul National University Boramae Medical Center during March 8–April 28, 2020. We extracted RNA from clinical specimens and detected SARS-CoV-2 by using the Allplex 2019-nCoV Assay kit (Seegene, http://www.seegene.com). We performed quantitation of the viral RNA with a standard curve constructed using in vitro transcribed RNA. This study was approved by the institutional review board at SMG-SNU Boramae Medical Center; written consent was waived.

We included 12 children in the study; 9 were mildly symptomatic and 3 were asymptomatic (Appendix Table 1). Median age was 6.5 years (range 27 days–16 years). Nasopharyngeal swab specimens tested positive for SARS-CoV-2 RNA in all 12 children, and 11 (92%) had positive RNA in their fecal specimens (Appendix Table 2). We collected saliva samples from 11 children; 8 (73%) tested positive.

Viral RNA load in the nasopharyngeal swabs peaked early at median 7.56 (range 6.19–10.56) log_10_ copies/mL and decreased over time (p<0.001 for trend) (Figure, panel A). The positivity of the specimens was 75% during week 2 and 55% during week 3 (Appendix Table 2). In comparison, the median initial fecal RNA load was 7.68 (range <4.10–10.27) log_10_ copies/mL and remained steadily high (p = 0.148 for trend) for >3 weeks (Figure, panel B). Fecal positivity remained >80%. The median RNA load in fecal samples was significantly higher than that for nasopharyngeal swab specimens during week 2 (7.26 vs. 6.19 log_10_ copies/mL; p = 0.006) and week 3 (7.61 versus 5.49 log_10_ copies/mL; p = 0.006). Except for 1 case, the RNA load in saliva declined rapidly with time (p = 0.003 for trend) (Figure, panel C). Positivity in saliva samples was 80% in week 1 but dropped sharply to 33% in week 2 and 11% in week 3.

**Figure:**
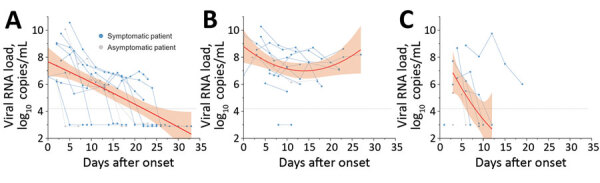
Changes in severe acute respiratory syndrome coronavirus 2 viral RNA load in A) nasopharyngeal swabs, B) feces, and C) saliva of mildly symptomatic and asymptomatic children with coronavirus disease over time, South Korea. The thick red line indicates trend in viral RNA load over time, and the shaded areas represent 95% CIs. The dashed line indicates the detection limit (1.25 × 10^4^ copies/mL). Specimens with undetectable viral RNA loads are shown under the dashed line. Days after onset indicates days after symptom onset for symptomatic patients, days after diagnosis for asymptomatic patients.

We collected urine specimens from the 12 patients after a median of 3 (range 0–8) days and plasma specimens at 2 (range 0–8) days from onset. Of these, urine samples of 2 (17%) patients tested positive (median load 5.69 [range 3.82–7.55] log_10_ copies/mL). Only 1 (8%) patient, 27 days of age, had RNA detected in plasma.

Symptomatic children had higher initial RNA load in nasopharyngeal swab specimens than asymptomatic children (9.01 vs. 6.32 log_10_ copies/mL; p = 0.048). We observed no significant differences in feces and in saliva and no correlation between RNA load and age.

In this study, we detected SARS-CoV-2 RNA in feces of 92% of mildly ill or asymptomatic children with COVID-19. In addition, the RNA load in feces remained steadily high, whereas that in nasopharyngeal swab specimens and saliva declined with time in both symptomatic and asymptomatic children. The detection of SARS-CoV-2 RNA in feces does not necessarily mean that infectious virus is present; thus, lack of virus isolation in our study limits interpretation in the context of infectivity. However, viable virus was isolated in feces in previous studies, and infectivity was dependent on viral RNA load (*3*,*5*,*6*). Considering these findings, proper handwashing when changing diapers in infants and adequate hygiene measures in restrooms are recommended to prevent the potential spread of the virus among household contacts.

Our findings also suggest that feces is a promising and reliable source for detecting both current and recent SARS-CoV-2 infection because the viral RNA is present in high loads for a prolonged time. Fecal specimens could aid in seeking the etiologic relationship between COVID-19 and unexpected manifestations in children.

We also detected SARS-CoV-2 RNA in saliva during the early phase of the infection for a short period of time. Live virus was isolated in saliva in a previous study, and the possibility of airborne transmission of the virus through normal speaking has been raised (*7*,*8*). Although the viral load in saliva drops rapidly, our findings suggest the necessity for children to wear masks, especially in schools, where children would talk in close proximity.

Appendix Additional information about the study of viral RNA load in children with COVID-19 in Seoul.
