# Supplementary material for: Viral RNA Load in Mildly Symptomatic and Asymptomatic Children with COVID-19, Seoul, South Korea
Source: Emerg Infect Dis. 2020 Oct;26(10):2497–9. doi: 10.3201/eid2610.202449 (PMC7510743; doi:10.3201/eid2610.202449)
Supplement: Appendix — Additional information about the study of viral RNA load in children with COVID-19 in Seoul. [file 20-2449-Techapp-s1.pdf]

Article DOI: https://doi.org/10.3201/eid2610.202449

Viral RNA Load in Mildly Symptomatic and Asymptomatic Children with COVID-19, Seoul

Appendix

Appendix Table 1. Clinical manifestations of 12 children with COVID-19, Seoul

Patient	Age/sex	Underlying disease	Symptoms	Pneumonia	O_2 supplement	SARS-CoV-2 RNA positive specimens	Outcome
1	27 d /F	None	Fever, cough, nasal stuffiness, vomiting	No	No	Nasopharyngeal swab, feces, saliva, urine, plasma	Improved
2	7 y /M	None	Fever	No	No	Nasopharyngeal swab, feces, saliva	Improved
3	10 y/M	None	Cough, sputum, sore throat, headache	No	No	Nasopharyngeal swab, feces, saliva	Improved
4	16 y/F	None	Chills, dizziness, cough, sputum, sore throat, nasal congestion	Yes	No	Nasopharyngeal swab, feces, saliva	Improved
5	14 y/F	None	Fever, cough, sputum, sore throat	No	No	Nasopharyngeal swab, feces, saliva	Improved
6	11 y/F	None	Cough, sputum, abdominal pain, diarrhea	No	No	Nasopharyngeal swab, feces (saliva not tested)	Improved
7	9 m/M	None	Fever, cough, rhinorrhea	No	No	Nasopharyngeal swab, feces, saliva	Improved
8	14 y/M	None	Headache, ageusia, anosmia	No	No	Nasopharyngeal swab	Improved
9	12 m/M	None	Cough	No	No	Nasopharyngeal swab, feces, urine	Improved
10	6 y/F	None	Asymptomatic	No	No	Nasopharyngeal swab, feces, saliva	Improved
11	5 y/F	None	Asymptomatic	No	No	Nasopharyngeal swab, feces	Improved
12	11 m/F	None	Asymptomatic	No	No	Nasopharyngeal swab, feces, saliva	Improved

Appendix Table 2. Positivity and viral RNA load of SARS-CoV-2 in nasopharyngeal swab, feces, and saliva in children with COVID-19*

	Nasopharyngeal swab (n = 12)		Feces (n = 12)		Saliva (n = 11)	
Category	No. positive/total (%)†	Median viral load, $\log_{10}$ copies/mL (range)	No. positive/total (%)	Median viral load, $\log_{10}$ copies/mL (range)	No. positive/total (%)	Median viral load, $\log_{10}$ copies/mL (range)
Total	12 (100)	6.29 (<4.10–10.56)	11 (92)	7.63 (<4.10–10.27)	8 (73)	5.50 (<4.10–9.75)
0–7 d after onset	12/12 (100)	7.38 (<4.10–10.56)	11/11 (100)	7.97 (6.07–10.27)	8/10 (80)	5.74 (<4.10–8.87)
8–14 d after onset	9/12 (75)	6.19 (<4.10–8.59)	11/12 (92)	7.26 (<4.10–8.89)	3/9 (33)	<4.10 (<4.10–9.75)
15–21 d after onset	6/11 (55)	5.49 (<4.10–6.95)	8/9 (89)	7.61 (<4.10–8.71)	1/9 (11)	<4.10 (<4.10–7.51)
>21 d after onset	3/11 (27)	<4.10 (<4.10–6.73)	4/5 (80)	7.57 (<4.10–8.61)	0	0

* COVID-19, coronavirus disease 2019; SARS-CoV-2, severe acute respiratory syndrome coronavirus 2.
†Positivity for SARS-CoV-2 was calculated from the number of patients with known test results.
